# Small head size and delayed body weight growth in wild Japanese monkey fetuses after the Fukushima Daiichi nuclear disaster

**DOI:** 10.1038/s41598-017-03866-8

**Published:** 2017-06-14

**Authors:** Shin-ichi Hayama, Moe Tsuchiya, Kazuhiko Ochiai, Sachie Nakiri, Setsuko Nakanishi, Naomi Ishii, Takuya Kato, Aki Tanaka, Fumiharu Konno, Yoshi Kawamoto, Toshinori Omi

**Affiliations:** 1 Nippon Veterinary and Life Science University, Tokyo, Japan; 2 Conservation and Animal Welfare Trust, Tokyo, Japan; 3 Fukushima Mirai Agricultural Cooperative, Fukushima, Japan; 4 Primate Research Institute, Kyoto University, Aichi, Japan

## Abstract

To evaluate the biological effect of the Fukushima Daiichi nuclear disaster, relative differences in the growth of wild Japanese monkeys (*Macaca fuscata*) were measured before and after the disaster of 2011 in Fukushima City, which is approximately 70 km from the nuclear power plant, by performing external measurements on fetuses collected from 2008 to 2016. Comparing the relative growth of 31 fetuses conceived prior to the disaster and 31 fetuses conceived after the disaster in terms of body weight and head size (product of the occipital frontal diameter and biparietal diameter) to crown-rump length ratio revealed that body weight growth rate and proportional head size were significantly lower in fetuses conceived after the disaster. No significant difference was observed in nutritional indicators for the fetuses’ mothers. Accordingly, radiation exposure could be one factor contributed to the observed growth delay in this study.

## Introduction

The Fukushima Daiichi nuclear power plant (NPP) disaster that occurred in March 2011 exposed a large number of humans and wild animals to radioactive substances. Several studies of wild animals in Fukushima investigated health effects of the disaster, such as morphological abnormalities in gall-forming aphids (*Tetraneura sorini*, *T*. *nigriabdominalis*)^1^ and pale grass blue butterfly (*Zizeeria maha*)^2^, hematological abnormalities in carp (*Cyprinus carpio*)^3^, and chromosomal aberrations in wild mice (*Apodemus argenteus*, *Mus musculus*)^4^. However, there is no research investigating long-term exposure to radiation on mammals that typically have long life-span to date. This study is the first report to observe long-term biological effects of the pre- and post-NPP disaster on non-human primates in Fukushima.

We previously studied radioactive exposure and its effect on health of Japanese monkeys (*Macaca fuscata*) inhabiting Fukushima City, which is located approximately 70 km from the Fukushima Daiichi NPP^5, 6^. After the NPP disaster, the range of radiocesium soil concentrations in Fukushima City was 10,000–300,000 Bq/m^2^. Hayama *et al*.^5^ investigated chronological changes in muscle radiocesium concentrations in monkeys inhabiting Fukushima City from April 2011 to June 2012. The cesium concentration in monkeys’ muscle captured at locations with 100,000–300,000 Bq/m^2^ was 6000–25,000 Bq/kg in April 2011 and decreased over 3 months to approximately 1000 Bq/kg. However, the concentration increased again to 2000–3000 Bq/kg in some animals during and after December 2011, before returning to 1000 Bq/kg in April 2012, after which it remained constant.

Fukushima monkeys had significantly lower white and red blood cell counts, hemoglobin, and hematocrit, and the white blood cell count in immature monkeys showed a significant negative correlation with muscle cesium concentration^6^. These results suggested that the short-term exposure to some form of radioactive material resulted in hematological changes in Fukushima monkeys.

The effects associated with long-term low-dose radiation exposure on fetuses are among the many health concerns. Children born to atomic bomb survivors from Hiroshima and Nagasaki showed low birth weight, high rates of microcephaly^7^, and reduced intelligence due to abnormal brain development^8^. Experiments with pregnant mice or rats and radiation exposure had been reported to cause low birth weight^9, 10^, microcephaly^11–13^, or both^14, 15^. We identified one similar study on wild animals^16^, which reported that the brains of birds captured in the vicinity of the Chernobyl NPP weighted lower compared to those of birds captured elsewhere.

The population of Japanese monkeys in Fukushima City had been systematically managed since 2008 according to a management plan based on law and regulated by Fukushima Prefecture to reduce damage to agricultural crops. Our research group studied the reproductive and nutritional status of the Japanese monkey population by performing autopsies on individuals captured and euthanized by Fukushima City^17^. These Japanese monkeys were the first wild primate population exposed to radiation as result of nuclear disaster. However, there was no other study either in Chernobyl or Fukushima that followed fetal development over time or compared fetal development before and after long-term radiation exposure in the same wild animal populations.

The objectives of this study were to compare changes in the fetal development of Japanese monkeys in Fukushima City before and after the NPP disaster to determine evidence of developmental delay in Japanese monkey fetuses.

## Results

Radiocesium was detected in mothers’ muscle that had conceived after the NPP disaster (Table 1). Mean muscle radiocesium concentration was 1059 Bq/kg for mothers that mated in 2011 and gave birth in 2012 (n = 14), although the concentration decreased gradually in subsequent years up to 22 Bq/kg for mothers that gave birth in 2016 (n = 3). Because muscle tissue was not available prior to the NPP disaster, muscle radiocesium concentrations for individuals captured pre-disaster could not be measured. However, muscle radiocesium concentrations in wild Japanese monkeys captured in 2012 in Aomori Prefecture, which is also located in the Tōhoku region 400 km north from the NPP, were below the detection limit^2^, therefore, we assumed that the muscle radiocesium concentrations in the Japanese monkeys in Fukushima City prior to the disaster were also below the detection limit.

**Table 1:** Muscle radiocesium concentration in pregnant Japanese monkeys according to birth year.

Birth year	Muscle radiocesium concentration in pregnant monkeys (Bq/kg)				
	*n*	Mean	SD	Min.	Max.
2012	14	1059	478	119	1959
2013	8	310	206	41	642
2014	4	331	154	102	431
2015	2	181	—	35	327
2016	3	22	19	15	32

Similarly, although the air dose in the area of Fukushima City inhabited by the Japanese monkeys was 1.1 to 1.2 µSv/h in April, 2011, it has decreased, reaching 0.10 to 0.13 µSv/h in May, 2016 (Table 2). Based on these measurements, it is estimated that monkeys in this area received accumulated air doses of at least 12 mSv over the five years since the NPP disaster.

**Table 2:** Air doses measured at monitoring sites maintained by Fukushima Prefecture near the study area (μSv/h).

Measurement date	Monitoring site (μSv/h)		Duration (days)	Mean accumulated dose (μSv)
	Iizaka tunnel	Ohzaso		
Apr. 2011	1.1	1.2	30	828
Aug. 2011	0.54	0.50	120	1498
Feb. 2012	0.5	0.46	180	2074
May 2012	0.51	0.35	90	1048
Oct. 2012	0.44	0.34	150	1404
May 2013	0.28	0.25	210	1336
May 2014	0.18	0.22	365	1752
May 2015	0.16	0.16	365	1402
May 2016	0.10	0.13	365	1007
Total				12349

Mean accumulated doses were estimated to multiply the average value of air doses in two monitoring site by the number of days between consecutive measurement dates.

**Figure 1:**
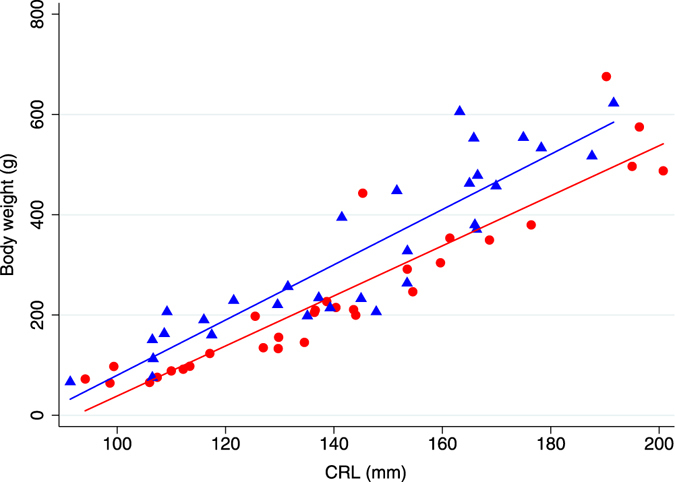
Body weight (g) as a function of CRL (mm) in Japanese monkey fetuses pre- and post-NNP disaster (n = 62). The figure shows regressions between body weight and CRL in pre- and post-disaster groups. Blue triangles were pre-disaster monkey fetuses with the blue line representing the fitted values (n = 31); R^2^ = 0.86. Red circles were post-disaster monkey fetuses with the red line representing the fitted values (n = 31); R^2^ = 0.82.

**Figure 2:**
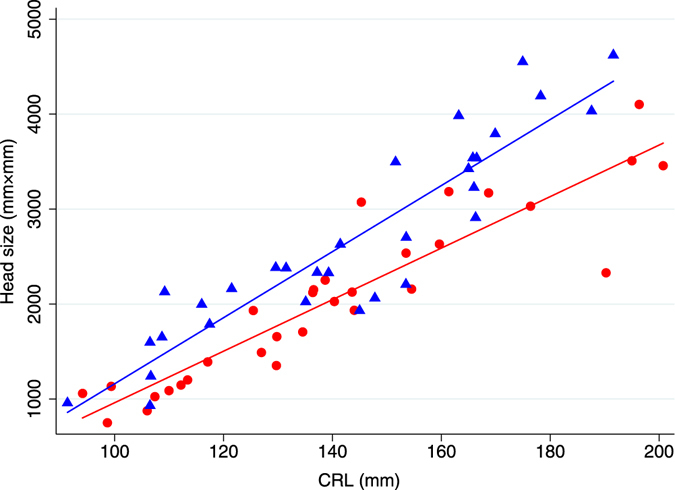
Head size (mm^2^) as a function of CRL (mm) in Japanese monkey fetuses pre- and post-NNP disaster (n = 62). The figure shows regressions between head size and CRL in pre- and post-disaster groups. Blue triangles were pre-disaster monkey fetuses with the blue line representing the fitted values (n = 31); R^2^ = 0.85. Red circles were post-disaster monkey fetuses with the red line representing the fitted values (n = 31); R^2^ = 0.84.

The descriptive statistics for Japanese monkey fetuses in Fukushima were shown in Table 3. The median body weight (g) and median body weight growth rate (g/mm) were significantly different between pre- and post-disaster groups (p = 0.032 and 0.0083, respectively). The mean biparietal diameter (mm), occipital frontal diameter (mm), head size (mm^2^), and proportional head size (mm) were significantly different between pre- and post-disaster groups (p = 0.046, 0.018, 0.014, and 0.0002, respectively). CRL was not significantly different between the two groups. Regression lines describing association of body weight and CRL in pre- and post-disaster groups were described in Fig. 1. Post-disaster regression line was significantly lower than pre-disaster regression line (p < 0.0001) (Table 4). Regression lines describing association of head size and CRL in pre- and post-disaster groups were described in Fig. 2. Post-disaster regression line was significantly lower than pre-disaster regression line (p < 0.0001) (Table 5).

**Table 3:** Descriptive statistics for pre- and post-disaster monkey fetuses in Fukushima (n = 62).

Variables	Pre-disaster (n = 31)	Post-disaster (n = 31)	P-value
Body weight (g) (median (range))	256.6 (66.5–622.5)	205.4 (64.3–675.5)	0.032
CRL (mm) (mean ± SD)	143.4 ± 27.0	140.2 ± 30.0	0.66
Occipital frontal diameter (mm) (mean ± SD)	55.9 ± 11.2	50.0 ± 12.2	0.046
Biparietal diameter (mm) (mean ± SD)	46.0 ± 9.4	40.2 ± 9.2	0.018
Head size (mm^2^) (mean ± SD)	2668.94 ± 1025.5	2051.6 ± 885.3	0.014
Body weight growth rate (g/mm) (median (range))	1.9 (0.7–3.7)	1.5 (0.6–3.6)	0.0083
Proportional head size (mm) (mean ± SD)	18.0 ± 4.2	14.1 ± 3.6	0.0002

**Table 4:** Multiple linear regression for body weight in Japanese monkey fetuses in Fukushima (n = 62).

Variables	Coefficient		95% CI (lower, upper)	P-value
	Estimative	Standard Error		
CRL	5.0	0.40	4.61, 6.41	<0.0001
Pre-disaster	1			
Post-disaster	10.5	87.08	−163.81, 184.82	0.904
Pre/Post-disaster*CRL	−0.5	0.60	−1.72, 0.69	0.393
Intercept	−471.2	65.2	−601.78, −340.59	<0.0001

R^2^ = 0.85; adjusted R^2^ = 0.84.

**Table 5:** Multiple linear regression for head size in Japanese monkey fetuses in Fukushima (n = 62).

Variables	Coefficient		95% CI (lower, upper)	P-value
	Estimative	Standard Error		
CRL	34.8	2.6	29.52, 40.0	<0.0001
Pre-disaster	1			
Post-disaster	566.4	506.2	−446.88, 1579.63	0.27
Pre/Post-disaster*CRL	−7.66	3.5	−14.66, −0.65	0.033
Intercept	−2318.9	379.2	−3077.96, −1559.76	<0.0001

R^2^ = 0.86; adjusted R^2^ = 0.85.

The body fat index for the mothers of these fetuses was not significantly different before and after the NPP disaster (Z = 1.213; P = 0.219).

## Discussion

Body weight and head size relative to the CRL were lower in fetuses conceived after the NPP disaster compared with fetuses conceived prior to the NPP disaster. Japanese monkeys in Fukushima City first conceive in fall when they were five years old and gave birth in spring when they were six years old^17^. Thus, we assumed that all the mothers we examined that conceived babies after the NPP disaster were continuously exposed to radiation from at the time of the disaster in 2011.

Growth retardation of the fetuses could be caused by the deterioration of the mothers’ nutritional status. However, we did not observe any difference in the body fat index of mothers pre- and post-NPP disaster. Therefore, the growth retardation of the fetuses was unlikely to be associated with to the mothers’ nutritional status. Other factors such as climate changes or food nutrient components might have affected the growth of fetuses. The limitations of this study were that we were not able to obtain samples to look at histological change that might have contributed to the cause of delayed fatal growth and the sample size were relatively small because of the nature of the sampling collection. It might have been ideal to compare monkeys from the evacuation order area to monkeys from the non-contaminated area of Fukushima; however, there was no other area such besides the one in this study that performed systematic large-scale capturing aimed at seizing hundreds of monkeys. In addition, there had been access limitations beyond the evacuation order area. For these reasons, it is impossible to replicate an equivalent study elsewhere at this time.

In experiments using mice and rats, radiation exposure has been reported to cause reduced fetal weight, microcephaly, and reduced brain mass^9–15^. However, most of these experiments involved exposing the mother to a single radiation dose at a fetal age of 10 days or later when the brain undergoes development. Such exposure may be qualitatively different from the low-dose, long-term exposure following an NPP disaster. The radiation doses in these experiments varied substantially. Hande *et al*.^9^ exposed mice to 9 mGy of 70 kilo-Volt peak X-rays at fetal ages of 3.5, 6.5, and 11.5 days, and found that birth weight was reduced relative to the control mice in all cases. Uma Devi *et al*.^15^ exposed mice to 0.25 Gy at a fetal age of 11.5 days and observed reduced head size at birth. In addition, they observed negative correlation between radiation dose and head size in fetuses exposed to 0.05 to 0.15 Gy.

The number of low birthweight children born to residents of some highly contaminated areas of Belarus increased between 1982 and 1990, after the Chernobyl NPP disaster^18^. Hujuel *et al*.^19^ conducted a longitudinal survey of women exposed to radiation through dental treatment who subsequently gave birth. They reported that women exposed to 0.4 mGy or more had increased risk (odds ratio 2.27) of giving birth to a child weighing 2500 g or less. Goldberg *et al*.^20^ elucidated the relationship between the level of radiation exposure as a result of medical exams prior to conception and birthweight, and found that birthweight decreased by 37.6 g for every cGy of exposure. Such medical exposure is believed to affect the mother’s gonads and endocrine glands rather than the fetus itself. There is still uncertainly to determine whether the retarded growth we observed was a direct effect of the radiation exposure.

Otake and Schull^8^ conducted a temporal variation study of mothers exposed to radiation by the atomic bombs in Hiroshima and Nagasaki. They did not observe any effect in newborns that had been exposed between fetal ages of 0 to 8 weeks, and the highest rates of microcephaly and other brain damage occurred in newborns exposed between fetal ages of 8 to 15 weeks. Given that the latter period was when the human brain undergoes rapid development, damage due to radiation exposure during this period might cause severe effect on fetuses.

The previous research suggested that the low birthweight and small head sizes observed in fetuses conceived after the NPP disaster were result of radiation exposure. However, we were not able to quantify the external and internal radiation dose in individual wild animals. Although radiocesium was detected in the muscles of all individuals captured after the NPP disaster, the cumulative exposure was unclear since the biological half-life of radiocesium in monkeys was approximately 3 weeks^5^. Furthermore, because of the small sample size, it was difficult to determine the causal relationship of exposure dosage and the effect on fetuses.

Although we showed that fetal proportional head size reduced after the NPP disaster, it was not possible to identify anatomically which part of the brain was developmentally retarded. Hossain *et al*.^12^ studied the brains of 6- to 12-month-old mice that were exposed to cobalt 60 at a fetal age of 14 days. Brain weight decreased at exposure rates of 0.5 to 1.5 Gy and the number of neurons in the hypothalamus in the CA3 region decreased significantly. We started to perform histological examination brain of fetuses and juvenile monkeys conceived after the NPP disaster to identify the regions of the brain that were developmentally retarded and the effect of retarded growth on post-natal development for further study.

## Methods

### Animals and ethics

Carcasses of Japanese monkeys were provided by Fukushima City. Monkeys were culled as a measure against crop damage with the permission of the governor of Fukushima Prefecture, according to the Fukushima Japanese Monkey Management Plan which was established based on the Wildlife Protection and Hunting Management Law. Monkeys were captured using box traps and killed with a gun by licensed hunters at the request of Fukushima City. The capture and killing method was in accordance with the guidelines of the management plan stated above and should not be an ethical concern. This killing method was also in accordance with guidelines published by the Primate Research Institute, Kyoto University^21^. The Japanese monkeys inhabiting this study area were not listed as an endangered species on the Japanese Red List, as revised by the Ministry of the Environment in 2012^22^.

### Fetuses and muscle samples

Fetuses were collected from pregnant Japanese monkeys from 2008 to 2016. Carcasses were transported under refrigerated conditions to our laboratory and subjected to autopsy. The body weight of each monkey was measured in grams. During the autopsy after the NPP disaster, 500–1000 g of muscle tissue was collected from the hind limbs to measure radiocesium content. Skeletal muscle was used because organs weighing 500 g or more were required to measure radiocesium concentration. The muscle tissue was stored frozen at −30 °C until it was used for radioactivity measurements.

After the fetuses were removed from the uterus during autopsy, body weight was measured to the nearest gram, and crown rump length (CRL), the length of the fetus from the top of its head to bottom of torso, was measured to the nearest millimeter. CLR was most commonly used as the somatometric measures for age assessment in physical and neurological examination^23^.

Fetuses were preserved in 10% neutral buffered formalin solution. The fetuses analyzed including specimens whose CRL was 90 mm or greater (fetal age of about 3 months or greater) and whose crania were ossified, allowing external measurement. Fetal head size was defined as the product of the biparietal diameter and occipital frontal diameter. The biparietal diameter was one of the basic biometric parameters used to assess fetal size, and maximum width of the head. The occipital frontal diameter should be measured on maximum length between forehead and occipital region. All specimens were measured by the same person, and to the nearest 0.1 mm using a caliper.

The Japanese monkeys in Fukushima City were seasonal breeders that bear young in March and April^17^. Accordingly, the fetuses collected in 2011 were almost fully developed at time of the NPP disaster. Thus, the fetuses were divided into those conceived before 2011 (pre-disaster) and those conceived in 2011 or later (post-disaster) and compared. The sample comprised 31 fetuses conceived pre- disaster and 31 fetuses conceived post-disaster.

### Fat index

During autopsy, fat indices were calculated to evaluate the monkeys’ nutritional status. In a previous study^24^, the ratio of mesenteric fat weight to body weight was proportional to the percentage of body fat in Japanese monkeys. The fat index was defined as mesenteric fat weight (g) divided by body weight (g), multiplied by 1000.

### Radioactivity measurements

Muscle radiocesium concentration was measured in 31 mothers that had conceived after the disaster. The radioactivity of radiocesium in the muscle samples was analyzed with a germanium semiconductor spectrometer (GC2020-7500SL-2002 CSL, Canberra, Meriden, CT) and a NaI (T1) scintillation detector (AT1320A, Atometex, Minsk, Belarus). Data were corrected to a background radiation dose in the measurement environment on an as-needed basis. ^134^Cs was detected using 604.70 and 795.85 keV gamma-rays, whereas ^137^Cs was detected using 661.6 keV gamma-rays. The radioactivity of radiocesium was adjusted to the value on the day of capture based on its physical half-life. The limit of detection was 10 Bq/kg. Muscle cesium concentration was calculated as the combined concentration of ^134^Cs and ^137^Cs per kilogram of fresh muscle.

To estimate the external exposure in monkeys, we used measurements taken 1 m above the ground at two air dose monitoring sites maintained by Fukushima Prefecture at Iizuka (N37°49′33.7″, E140°26′52.8″) and Ohzaso (N37°47′11.6″, E140°24′10.8″) near the monkeys’ habitat in Fukushima City. Between April 2011, just after the NPP disaster, and May 2016, Fukushima Prefecture performed nine air dose measurements at these monitoring sites^25^. Mean accumulated external exposure doses were estimated to multiply the average value of air doses in two monitoring site by the number of days between consecutive measurement dates.

### Statistics

All the monkeys who were undergoing fetal growth prior to the disaster were categorized in one “unexposed” group (pre-disaster), and those who underwent fetal growth after the disaster were classified as “exposed” (post-disaster) because there was no misclassification of this measure of exposure. The Shapiro-Wilk test was used to check for normality of each variable. The Student’s t-test was used to compare CRL, biparietal diameter, occipital frontal diameter, and head size to see if there was any difference between pre- and post-disaster groups, and the Wilcoxon-Mann-Whitney test was used to compare body weight between groups. Body weight growth rate was determined by using body weight divided by CRL, and the proportional head size was determined by using head size divided by CRL. The Student’s t-test was performed to compare the body weight growth rate and the Wilcoxon-Mann-Whitney test was performed to compare the proportional head size between pre- and post-disaster groups. Regression lines were generated describing the association of body weight and CRL, and head size and CRL. Multiple linear regressions were performed with dependent variables (body weight and head size) and explanatory variables (CRL and pre/post disaster) with an interaction term (pre/post disaster*CRL). Models were fit for each regression and tested using likelihood ratio test with and without interaction.

Stata/IC 13.1 (Stata Corp LP, College Station, Texas USA) was used for all analyses. For statistical estimation and inferences, two-sided hypothesis tests were used with a 5% significance level.

Significant differences in fat index were evaluated by the Wilcoxon rank-sum test.
